# IgG4-related ophthalmic disease masquerading as ciliary body tumors and scleritis in both eyes: a case report

**DOI:** 10.1186/s12886-023-02822-7

**Published:** 2023-03-09

**Authors:** Jiayue Ma, Manyun Xie, Kejun Long, Mi Deng, Liang Zhou, Jing Luo

**Affiliations:** 1 Department of Ophthalmology, The Second Xiangya Hospital, Central South University, No. 139, Renmin Middle Road, Furong District, Changsha, 410011 China; 2 Hunan Clinical Research Center of Ophthalmic Disease, Changsha, China

**Keywords:** Immunoglobulin G4-related ophthalmic disease, Autoimmune disease, Ophthalmic imaging, Ocular tumor, Scleritis, Cytokine level

## Abstract

**Background:**

To report a rare case of IgG4-related ophthalmic disease (IgG4-ROD) manifesting as intraocular masses and scleritis in both eyes in a 61-year-old male and to investigate the changes in multimodal imaging features of the lesion sites and helper T-cell type 1 (Th 1)/Th 2/Th 17 cytokine levels in the aqueous humor.

**Case presentation:**

A patient with IgG4-ROD seemingly manifested with an intraocular tumor in the left eye and sequentially, with an inflammatory mass in the ciliary body and scleritis in the right eye. The patient complained of vision loss of 6 months duration in the left eye at his first visit. With a preliminary diagnosis of an intraocular tumor, enucleation of the left eyeball and histopathological examination were performed. Approximately 3 months later, the patient started to experience headache, eye pain, and declining vision in the right eye. Ophthalmic imaging revealed a ciliary mass and scleritis. Th 1/Th 2/Th 17 cytokine levels and multimodal imaging findings were analyzed before and after corticosteroid treatment. Histopathological examination and immunohistochemistry (IHC) of the enucleated left eye demonstrated lymphoplasmacytic infiltration with an IgG4+/IgG+ cell ratio of approximately 40%, pointing to the diagnosis of probable IgG4-ROD. Long-term treatment with corticosteroids led to significant improvement in the signs and symptoms of the left eye. Th 1/Th 2/Th 17 cytokine profile monitoring of the aqueous humor and multimodal imaging of the right eye showed gradual regression of the mass and attenuation of ocular inflammation during treatment.

**Conclusions:**

Patients with an atypical presentation of IgG4-ROD, such as intraocular masses and scleritis, are likely to experience a significant delay in diagnosis. This case demonstrates the significance of IgG4-ROD in the differential diagnosis of intraocular tumors and ocular inflammation. IgG4-RD is a newly diagnosed disease with multi-organ involvement and little is known about its pathogenesis, particularly in the eye. The present case will open new challenges in the clinico-pathological diagnosis and research of this disease. Combined investigations of multimodal imaging and cytokine level detection of intraocular fluid provide a new and effective way to monitor disease progression.

## Background

Immunoglobulin G4-related disease (IgG4-RD) is characterized by the infiltration of IgG4-immunopositive plasmacytes, fibrosis, or mass formation in the involved organs and is usually combined with elevated serum IgG4 levels [1]. Comprehensive diagnostic criteria for IgG4-RD were established in 2011 [2]. As a relatively new and uncommon clinical entity, IgG4-RD may affect almost any organ and is considered an immune-mediated condition [1]. The typical ophthalmic manifestation of IgG4-RD is lacrimal gland enlargement in the form of dacryoadenitis. However, IgG4-related ophthalmic lesions are not limited to the lacrimal glands, but may also manifest with various lesions in the orbital and ocular tissues, such as the extraocular muscles, orbital nerve, and eyelid [3, 4]. Lesions in the ciliary body [5], sclera [6] and conjunctiva [7] have also been reported. The diagnostic criteria for IgG4-related ophthalmic disease (IgG4-ROD) have recently been reported (Table 1) and are based on both the clinical and histopathologic features of the ocular lesions [8]. Based on the diagnostic criteria, some conditions previously diagnosed as Mikulicz disease and various types of lymphoplasmacytic infiltrative disorders are considered as IgG4-ROD confirmed in recent studies [9]. Here, we report a rare case of IgG4-ROD presenting as masses in bilateral ciliary body and scleritis in both eyes. We also describe the dynamic changes of the mass and ocular inflammation by using multimodal imaging and cytokine monitoring of the aqueous humor during the course of treatment.

**Table 1:** Diagnostic criteria for immunoglobulin G4-related ophthalmic disease (IgG4-ROD), 2014

(1) Imaging studies show enlargement of the lacrimal gland, trigeminal nerve, or extraocular muscle as well as masses, enlargement, or hypertrophic lesions in various ophthalmic tissues (2) Blood test shows elevated serum IgG4 (≧135 mg/dl) (3) Histopathologic examination shows: A. Marked lymphocyte and plasmacyte infiltration, and sometimes fibrosis B. A germinal center is frequently observed C. Infiltration of IgG4+ plasma cells: ratio of lgG4+/IgG+ cells ≧40% or ≧50 IgG4+ plasma cells per high power field Definite IgG4-ROD: (1) + (2) + (3) Probable IgG4-ROD: (1) + (3) Possible IgG4-ROD: (1) + (2)	

## Case presentation

**Fig. 1:**
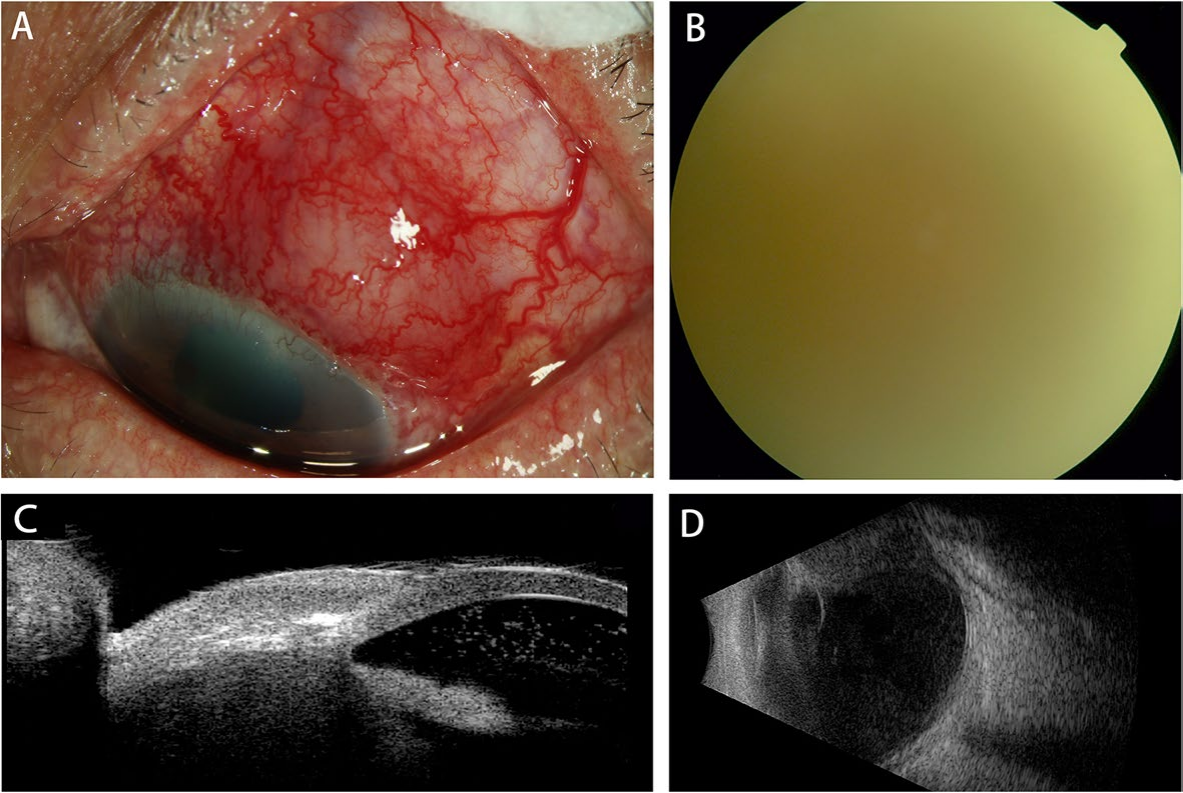
Multimodal imaging at the patient’s first visit before enucleation of the left eyeball. **A** Edema and severe vascular congestion of supratemporal conjunctiva and sclera tissue were observed in the slit-lamp examination. **B** A blurred fundus due to severe vitreous opacity was observed. **C** Ultrasound biomicroscopy (UBM) showed a mass in the superior-temporal part of the ciliary body and exudation in anterior chamber in the left eye. **D** B-ultrasonography showed a ciliary body mass with adjoining exudative retinal detachment in the left eye

**Fig. 2:**
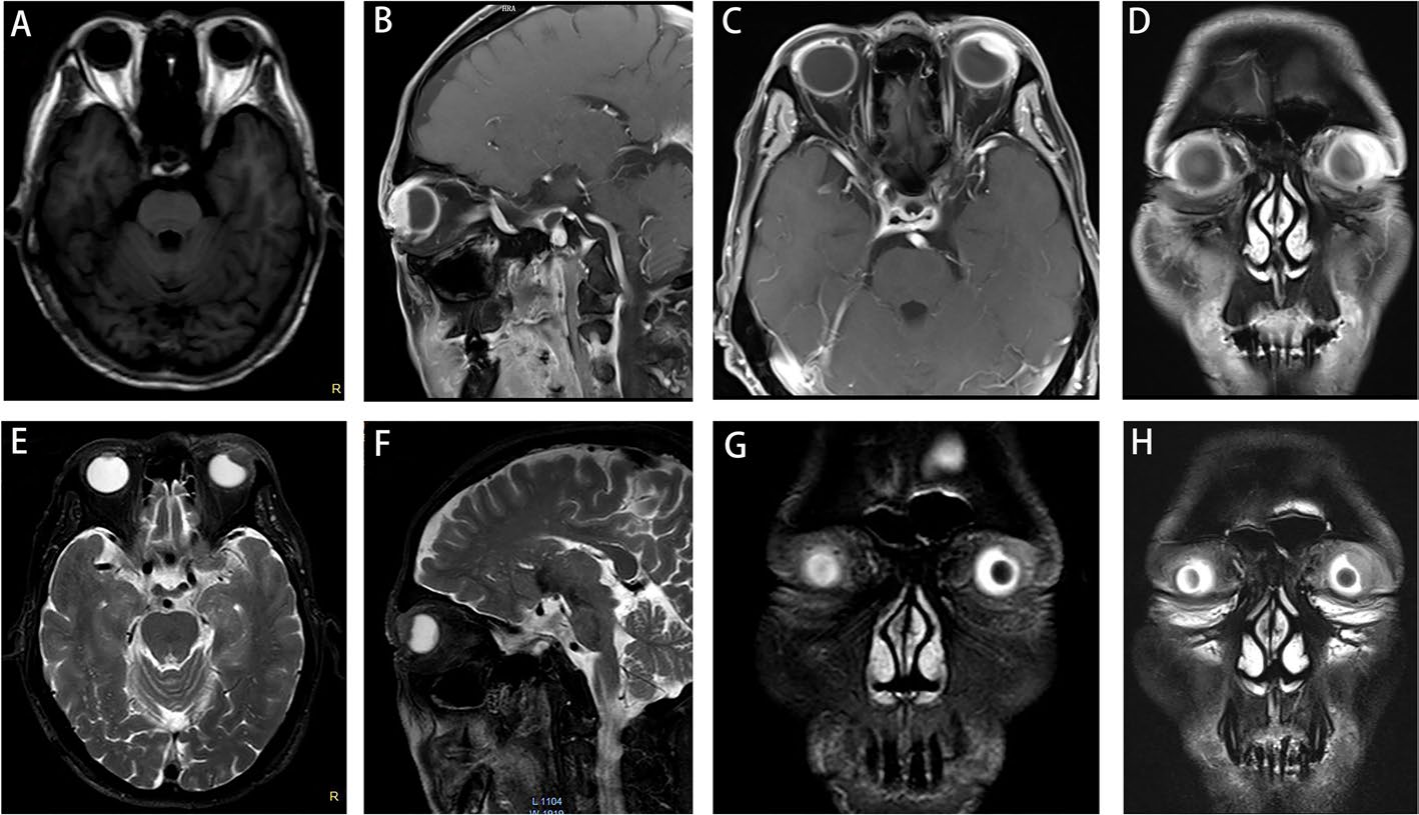
Magnetic resonance imaging (MRI) images of the left orbit at the patient’s first visit before enucleation of the left eyeball. **A** The lesion located in the ciliary body region of the left eye appeared isointense or slightly hyperintense on T1-weighted image (T1WI). **B**-**D** In T1WI post gadolinium enhancement MRI, the contrast enhanced scan showed enhancement in the lesion. The enhanced images manifested hyperintense on T1-f12d fat suppression (fs) (**B**: T1-f12d-fs-sag; **C**: T1-f12d-fs-tra; **D**: T1-f12d-fs-cor). **E**-**H** The lesion located in the ciliary body region of the left eye appeared hypointense on T2-weighted image (T2WI) (**E**: T2WI-tra; **F**:T2WI-sag; **G**: T2WI-cor). **H** The images manifested hypointense and more clear on T2-tse-fs (**H**)

A 61-year-old man was referred to the Department of Ophthalmology at the Second Xiangya Hospital, Central South University, in August 2018, and reported a 6-month history of vision loss in the left eye. He denied any history of surgery or trauma in the left eye. His best corrected visual acuity (BCVA) was 20/20 in the right eye and suspicious light perception (LP) with faulty light projection in the left eye. His intraocular pressures were 15 mmHg OD and 16 mmHg OS. Slit-lamp examination revealed edema and severe vascular congestion of the supratemporal conjunctiva and sclera, together with slight corneal edema, prominent keratic precipitates (KP), anterior chamber (AC) flare, a few inflammatory cells (++) in the AC, severe posterior synechia, and posterior capsule opacification in the left eye (Fig. 1A). Severe vitreous opacity resulting in a blurred fundus was also observed (Fig. 1B). Ultrasound biomicroscopy (UBM) showed a mass in the superior-temporal part of the ciliary body area of the left eye, with adjacent sclera and conjunctiva involvement (Fig. 1C). B-scan ultrasonography of the left eye revealed a highly reflective ciliary body mass accompanied by severe vitreous opacity and adjoining exudative retinal detachment in the left eye (Fig. 1D). Except for a mild lens opacity, no significant findings were observed in the right eye. Brain and orbit magnetic resonance imaging (MRI) revealed an intraocular mass in the ciliary body area. The lesion appeared isointense or slightly hyperintense on T1-weighted image (T1WI), and hypointense on T2-weighted image (T2WI). Contrast-enhanced scanning showed a progressive enhancement of the lesion. Ciliary body melanoma or schwannoma could not be excluded based solely on the MRI findings. MRI findings also revealed an abnormal signal suggestive of retinal detachment in the left eye (Fig. 2). The helper T-cell type 1 (Th 1)/ Th 2/Th 17 cytokine profile (IL-2, IL-4, IL-6, IL- 10, TNF-α, IFN-γ and IL-17A) in the aqueous humor of the left eye (Table 2) was also assessed and revealed a bursting increase of proinflammatory IL-6 (6969 pg/ml) level and slight to moderate increases of IL-4 (5.8 pg/ml), IL-10 (24.3 pg/ml), TNF-α (10.9 pg/ml) and IL-17A (29.1 pg/ml). A complete blood count (CBC) and urine and stool routine tests were unremarkable. Kidney and liver function, coagulation function, and antibodies against HIV, HBV, and syphilis also revealed normal results. A clinical diagnosis of an intraocular tumor, presumably ciliary body melanoma or schwannoma, with adjoining exudative retinal detachment (ERD) in the left eye was made.

**Table 2:** The helper T-cell type 1 (Th1)/Th2/Th17 cytokine profile in the aqueous humor of the left eye pre-operation

Cytokines	Cytokine levels (pg/ml)	Normal value (pg/ml)
IL-2	0.07	0–5.71
IL-4	5.8 ↑	0–2.8
IL-6	6969↑	0–5.3
IL- 10	24.3 ↑	0–4.91
TNF-α	10.9 ↑	0–2.31
IFN-γ	3.2	0–7.42
IL- 17A	29. 10↑	0–12.86

↑ means that the cytokine levels are higher than normal value

**Fig. 3:**
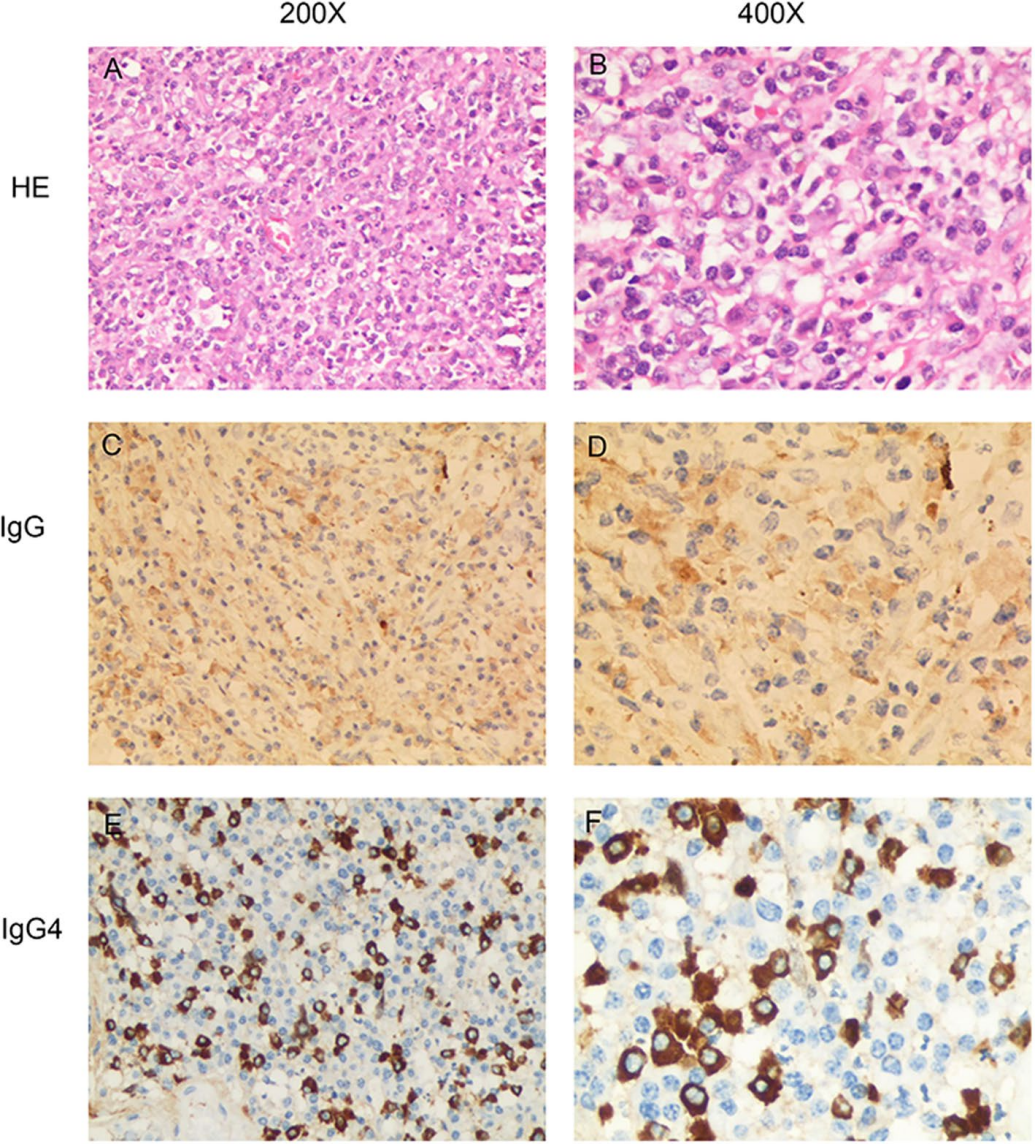
Histological and immunohistochemical findings of the left eye. **A** and **B** HE staining showed infiltration of plasmacytes, lymphocytes, neutrophils and histocytes, with proliferation of myofibroblasts. **C**, **D**, **E** and **F** Immunohistochemical staining showed that the plasmacytes were positive for IgG and IgG4, and the IgG4^+^/IgG^+^ plasma cell ratio was about 40%. (HE: hematoxylin-eosin staining)

The patient refused any other auxiliary examinations for financial reasons. Based on the information gathered from the slit-lamp examination, indirect binocular ophthalmoscopy, cytokine levels in the aqueous humor and the multimodal imaging mentioned above, a possible malignancy cannot be excluded. A joint physician-patient decision of enucleation of the left eyeball was made. The eyeball specimen was sent to the ocular pathology laboratory for histopathological analysis. Upon gross examination, a mass measuring (8 × 8 × 10 mm) was observed in the ciliary body area. Adjoining the ciliary body mass, ERD was noted. Microscopic examination revealed infiltration of plasmacytes, lymphocytes, neutrophils, and histocytes with proliferation of myofibroblasts. Immunohistochemical staining showed that the plasmacytes were positive for IgG and approximately 40% of these cells were positive for IgG4 (Fig. 3). Staining for Vimentin, Kappa, Lambda and CD68 was positive. CD3, SMA, LCA, CD20, and CD38 staining was focal or scattered positive. CK, EMA, S100, SOX- 10, HMB45, Melan-A, CD30, and ALKp80 staining were negative, and Ki67 staining showed 10% positivity (Fig. 3). Based on these histopathological findings, the patient was diagnosed with probable IgG4-ROD. Ideally, serum IgG4 levels should be evaluated to make a more precise diagnosis according to the diagnostic criteria for IgG4-ROD (Table 1). Moreover, given the high rate of extra-ophthalmic involvement, systemic examination and imaging (head, neck, chest, abdomen, and pelvis) are strongly recommended to identify other potential sites of involvement. However, the patient refused further systemic workup due to financial reasons.

**Fig. 4:**
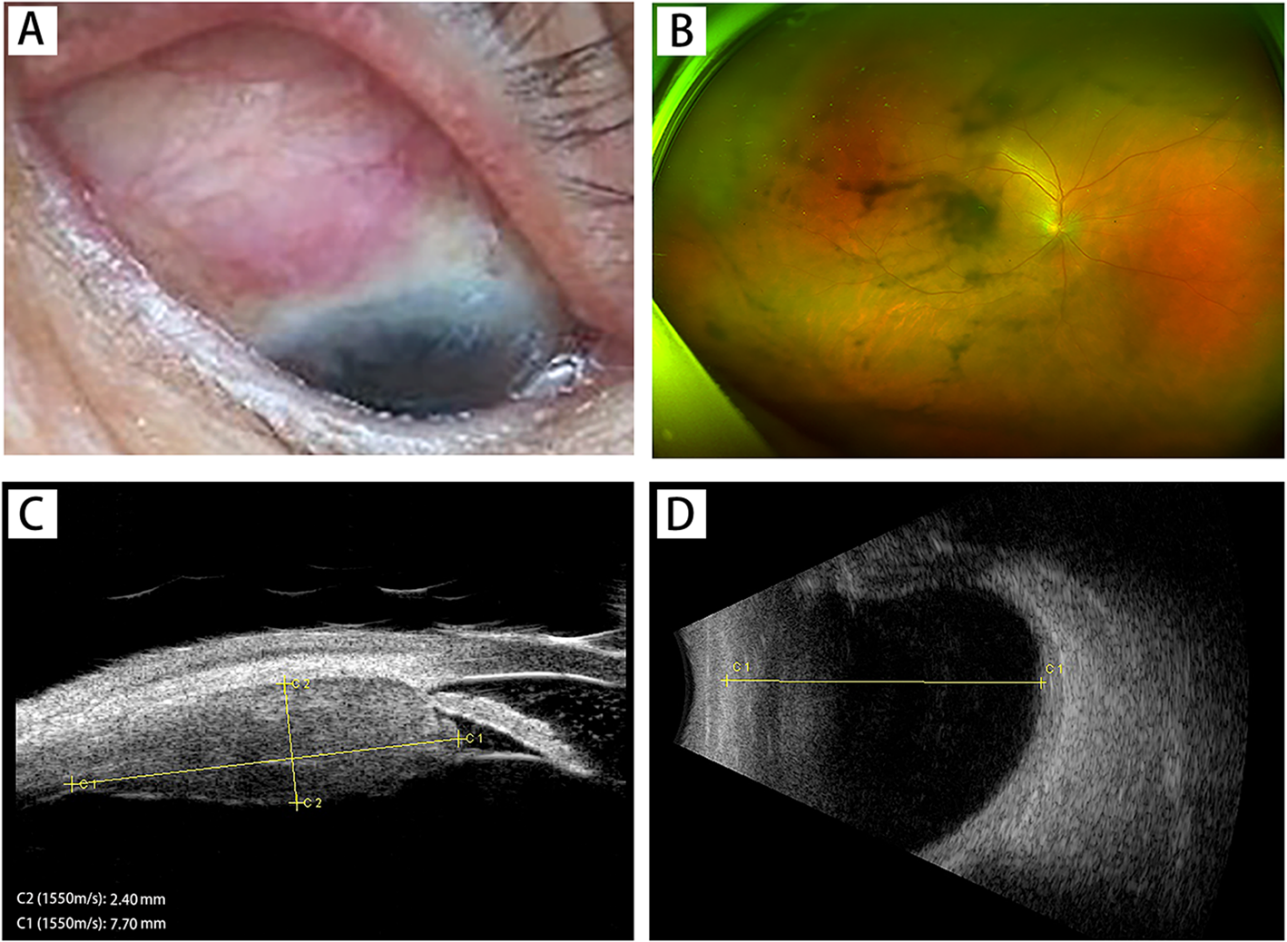
Multimodal imaging of the right eye pre-treatment. **A** Edema and vascular congestion of supratemporal conjunctiva and sclera tissue were observed in the slit-lamp examination. **B** Fundus examination showed a grey white mass located on the supratemporal peripheral fundus. Moderate vitreous opacity was present. **C** Ultrasound biomicroscopy (UBM) showed a mass measuring 7.7 mm × 2.4 mm in the superior-temporal part of the ciliary body and exudation in anterior chamber. **D** B-ultrasonography showed a ciliary body mass

**Fig. 5:**
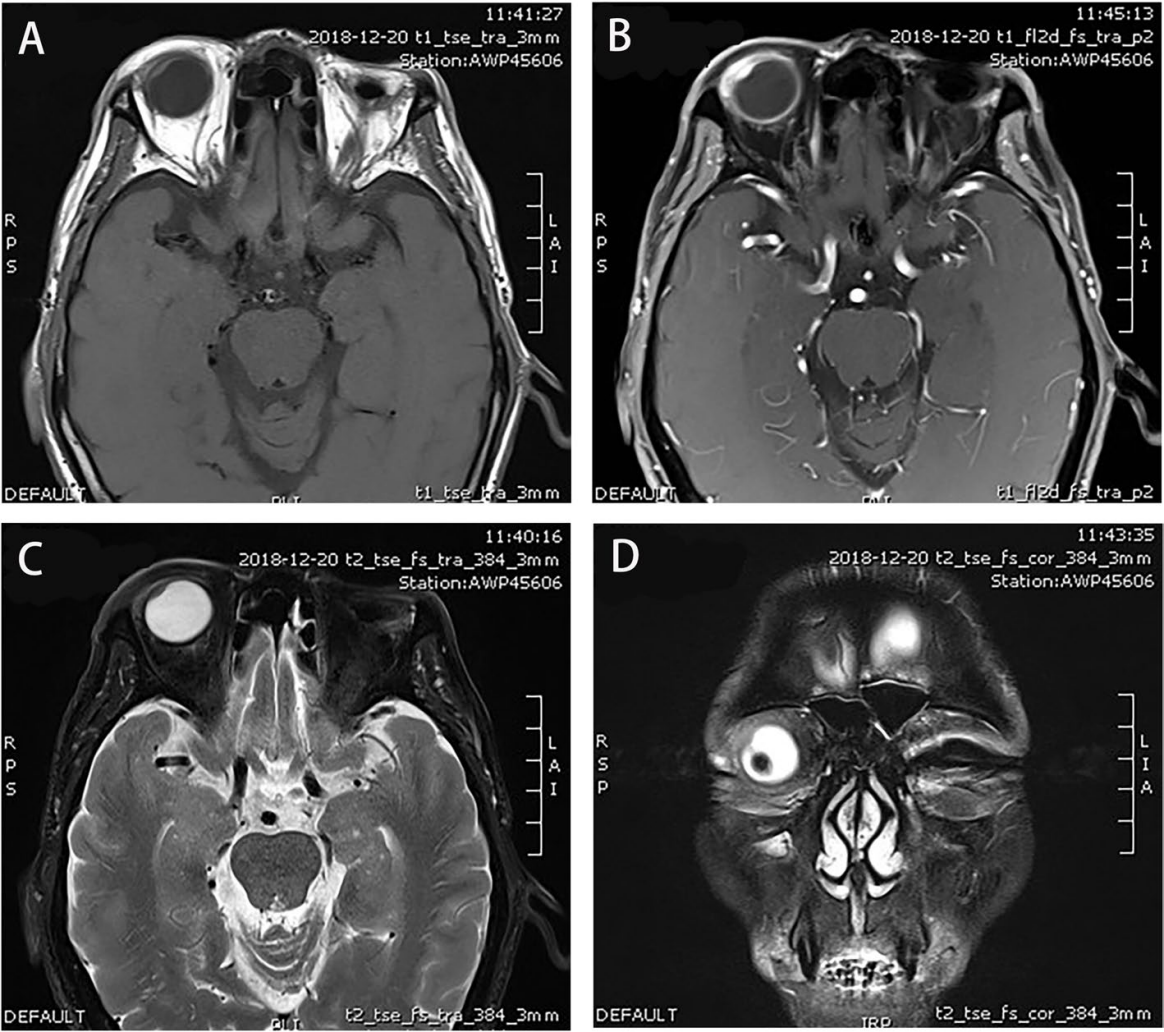
Magnetic resonance imaging (MRI) images of the right orbit at the patient’s second visit. **A** The lesion located in the ciliary body region of the right eye appeared isointense or slightly hyperintense on T1-weighted image (T1WI). **B** In T1WI post gadolinium enhancement MRI, the contrast enhanced scan showed enhancement in the lesion on T1-f12d fat suppression (fs). **C** and **D** The lesion located in the ciliary body region of the right eye appeared hypointense on T2-weighted image (T2WI) (C: T2-tse-fs-tra; D: T2-tse-fs-cor)

Four months later, the patient was referred to our clinic, complaining of eye pain and vision decline in the right eye and headache for 1 month, his BCVA was 12/20 in the right eye, and his intraocular pressure was 11 mmHg OD. Slit-lamp examination of the right eye revealed similar changes to the left eye 4 months ago. Prominent KP, slight AC flares, and moderate vitreous opacity were also observed in the right eye. Fundus examination revealed a gray-white mass located on the supratemporal peripheral fundus. The details of the mass could not be evaluated fully because of the moderate vitreous opacity. No other significant retinal abnormalities were observed on indirect binocular ophthalmoscopy. B-ultrasonography suggested a mass with high acoustic reflectivity in the supratemporal peripheral fundus. UBM showed a mass in the superior-temporal part of the ciliary body measuring 7.7 mm × 2.4 mm, with a subconjuntival lesion (Fig. 4). Brain and orbital MRI revealed lesions with abnormal signals located in the eyelid, peri-orbital tissue, supratemporal part of the wall of the eyeball, ciliary body area, and lacrimal gland of the right eye. The lesions also appeared isointense or slightly hyperintense on T1WI, hypointense on T2WI, and were enhanced on contrast-enhanced scan (Fig. 5). No other related findings in the brain were observed on the MRI scans. Th 1/Th 2/Th 17 cytokine (IL-2, IL-4, IL-6, IL-10, TNF-α, IFN-γ, and IL-17A) analysis in the aqueous humor of the right eye (Table 3) was assessed and revealed similar changes to the left eye 4 months ago, with a bursting increase of IL-6 (5773.61 pg/ml). An extensive work-up excluded infectious (including tuberculosis), rheumatic, inflammatory, and neoplastic causes. Both serum and aqueous humor IgG4 levels were within the normal ranges. MRI scan of the sacroiliac joint and the adjacent tissue, cervical and abdominal ultrasonography, and lung CT scanning showed no related abnormalities. Based on the findings and the medical history of the left eye, a clinical diagnosis of probable IgG4-ROD of the right eye was proposed.

**Table 3:** The helper T-cell type 1 (Th1)/Th2/Th17 cytokine profile in the aqueous humor of the right eye pre-treatment

Cytokines	Cytokine levels (pg/ml)	Normal value (pg/ml)
IL-2	1.5	0–5.71
IL-4	3.76↑	0–2.8
IL-6	5773.61↑	0–5.3
L- 10	11.36↑	0–4.91
TNF-α	2.87↑	0–2.31
IFN-γ	2.99	0–7.42
IL- 17A	19.4 ↑	0–12.86

↑ means that the cytokine levels are higher than normal value

**Fig. 6:**
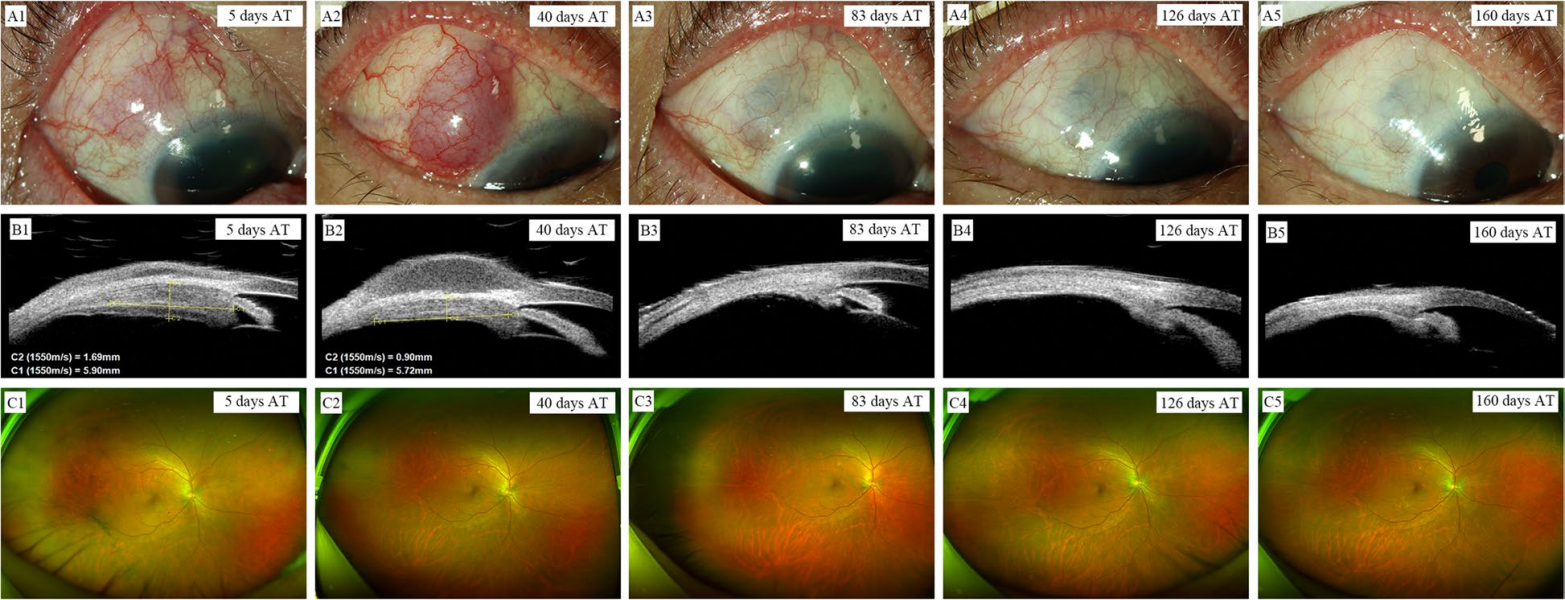
Changes of the right eye showed by multimodal imaging monitoring and IL-6 level after treatment (AT). **A1**-**A5** Slit-lamp examination showed relief of edema and congestion of conjunctiva and sclera at 5 days AT. A bulbar conjunctival swelling was observed at 40 days AT and only hyperpigmentation at the lesion location was observed at 83 days AT and remained stable as observed at the following timepoints. **B1**-**B5** UBM showed the gradual shrinking of the mass and the subconjunctival fluid during treatment. The subconjunctival fluid and the mass disappear completely at 83 days AT and at the following timepoints. **C1**-**C5** Wide-angle fundus photography showed that the grey white mass located on the supratemporal peripheral fundus cannot be detected since 40 days AT and gradual improvement of vitreous opacity was also observed

The patient was treated with 500 mg intravenous methylprednisolone for the first 7 days, followed by oral methylprednisolone (50 mg/day). Peribulbar injections of 5 mg dexamethasone once and prednisolone acetate (1%, Pred Forte) every 2 hours while awake were also administered to the right eye. The mass and intraocular inflammation gradually attenuated in the follow-ups by serial monitoring using slit lamp photography, UBM, B-ultrasonography, wide-angle fundus photography (Fig. 4), and aqueous humor Th 1/Th 2/Th 17 cytokine levels (Table 4). Notably, as the oral and topical steroids were tapered, prominent bulbar conjunctival swelling was observed in the lesion location 40 days after treatment (the dosage of oral steroid was 30 mg/day). UBM showed gradual accumulation of subconjunctival fluid, which explained the bulbar conjunctival swelling, while the mass size gradually decreased (Fig. 6B2 and B3). Aqueous humor cytokine analysis showed IL-6 levels were down-regulated to 1021.85 pg/ml at 9 days post-treatment (the dosage of oral steroid was 50 mg/day), and then rose gradually to 2981.5 pg/ml at 40 days post-treatment (the dosage of oral steroid was 30 mg/day), in accordance with the gradual subconjunctival fluid accumulation. Continuous corticosteroid treatment yielded prominent improvement, with gradual absorption of the subconjunctival fluid and shrinkage of the mass. At 11 weeks post-treatment, the dose of oral steroid was reduced to 5 mg/d as a maintenance dose. After nearly 3 months of corticosteroid treatment, all symptoms of the right eye resolved and remained stable at the following time points (Fig. 6). IL-4, IL-10, TNF-α, and IL-17A levels were readjusted to normal and IL-6 level decreased to nearly normal (20.72 pg/ml). BCVA of the right eye was 16/20.

**Table 4:** Changes of the helper T-cell type 1 (Th1)/Th2/Th17 cytokine levels in the aqueous humor of the right eye after treatment (AT)

Cytokines	9 days AT (pg/ml)	40 days AT (pg/ml)	3 months AT (pg/ml)	Normal value (pg/ml)
IL-2	0.95	1.43	1.01	0–5.71
IL-4	2. 13	2. 13	1.37	0–2.8
IL-6	1021.85↑	2981.5 ↑	20.72↑	0–5.3
IL- 10	2.32	1.24	0.66	0–4.91
TNF-α	0.83	0.95	1.06	0–2.31
IFN-γ	1.73	1.82	0.75	0–7.42
IL- 17A	31.74↑	7.75	3.04	0–12.86

## Discussion and conclusions

The frequency of IgG4-ROD ranges from 4 to 34% in IgG4-RD patients according to the largest case series published to date [10]. According to recent reports, IgG4-ROD is an emerging cause of uveitis, uveal masses, and scleritis with or without conjunctival infiltration and should be considered in any patient with multisystem inflammatory disease [3]. IgG4-ROD can mimic several infectious, inflammatory, and malignant disorders. More common causes of lacrimal gland masses, other orbital space-occupying lesions, scleritis, and uveitis should be ruled out before suspecting a diagnosis of IgG4-ROD [3].

In cases of orbital or intraocular masses, it is important to differentiate IgG4-ROD from malignant tumors (e.g. melanoma, lymphoma) and similar diseases [3, 5]. In our case, the MRI of the patient was quite similar to that of melanoma. Choroidal melanoma typically displays a high-intensity signal on T1-weighted images and a low signal intensity on T2-weighted images. Nevertheless, imaging findings may vary based on the degree of pigmentation and the presence of areas of necrosis or cavitation [11]. After gadolinium administration, the images showed intermediate-to-hyperintensity enhancement on T1WI [12]. At present, there is no established uniform description of the imaging findings for IGg4-ROD. IGg4-ROD has various imaging manifestations, including lacrimal gland type, ocular muscle type, mass type and diffuse type. In a study of Xiaoyan Wang, all types of IGg4-ROD were isointense or slightly hypointense on T1WI and slightly hyperintense on T2WI. The lacrimal gland type showed an obviously high signal on T2WI with fat suppression (fs). Moreover, if lymphoplasma cells and lymphoid follicles were predominant in the lesion, it appeared isointense or slightly hyperintense on T2WI. When fibrotic components were predominant, the lesion exhibited slight hypo-intensity on T2WI [13]. However, in another study of Lin Fu, the lacrimal gland type was found to be slightly hypointense on T1WI and isointense or slightly hypointense on T2WI. Contrast-enhanced imaging demonstrated a lesion with heterogeneous enhancement. The ocular muscle type, which is characterized by diffuse thickening of ocular muscles, presents blurred edges, slightly hyperintense T1WI, and marked reinforcement after enhancement [14]. In our case, we found that the lesion, which was located in the ciliary body area, appeared isointense or slightly hyperintense on T1WI, and hypointense on T2WI. Contrast-enhanced scanning showed progressive enhancement of the lesion. Therefore, we could not rule out the diagnosis of a malignant tumor by MRI before histopathological examination.

A long delay in the diagnosis of IgG4-RD remains common and may be responsible for irreversible sequelae due to extensive fibrosis [15]. Most reported cases of intraocular involvement have been unilateral. However, in the present case, both eyes were involved sequentially. The left eye was first involved and presented as a large intraocular mass and severe ocular inflammation, together with ERD, which displayed well on ophthalmic imaging including MRI, slit lamp photography, UBM, B-ultrasonography, and wide-angle fundus photography. Cytokine profile analysis showed a severe intraocular “inflammatory storm” characterized by an explosive rise of proinflammatory IL-6 together with increases in other tested inflammatory cytokines including IL-4, IL-10, TNF-α, and IL-17A. Immunohistochemistry (IHC) staining of the enucleated eyeball showed that the ratio of lgG4+/IgG+ cells was approximately 40%. The lymphoma and other ocular tumors were negative. Based on the diagnostic criteria for IgG4-ROD, the patient was diagnosed with probable IgG4-ROD. Lesions with similar features to the left eye (a ciliary body mass with ocular inflammation showed by ophthalmic imaging and cytokine analysis) were detected later in the right eye in the same patient, and systematic and local corticosteroids yielded satisfactory results, which further supported the diagnosis.

With advances in technology, several novel imaging modalities are available. Each modality has its pros and cons as well as its limitations. A combination of multiple imaging techniques can overcome these individual weaknesses and provide a comprehensive picture. Multimodal imaging is the concept of “bundling” images obtained from various imaging modalities that enable accurate localization and in vivo near-histologic assessment of ocular tissue, greatly aiding the clinician in establishing a diagnosis and monitoring the therapeutic response of ocular lesions. In the present case, we combined techniques including MRI, slit lamp photography, UBM, B-ultrasonography, and wide-angle fundus photography to evaluate ocular lesions. Notably, the bulbar conjunctival swelling observed by slip lamp in the lesion location 40 days after treatment might be easily considered as a sign of aggravation. However, as a powerful ophthalmic imaging tool for detecting anterior segment lesions of the eye [16], UBM clearly depicts the location and dynamic accumulation of the subconjunctival fluid and the gradual shrinking of the mass, helping ophthalmologists to develop a proper therapeutic strategy.

Cytokines play essential roles in inflamed eyes. Elevated aqueous humor concentrations of cytokines have been reported for different types of uveitis, and diverse cytokine profiles have been shown to be characteristic of specific diseases [17]. Consequently, cytokine patterns may serve as diagnostic and prognostic monitoring tools for clinicians, but are also useful for understanding the underlying immunopathogenic mechanisms. The expression patterns of these cytokines in IgG4-RD involving ocular tissues have not been investigated. We analyzed Th1/Th 2/Th17 cytokine (IL-2, IL-4, IL-6, IL-10, TNF-α, IFN-γ and IL-17A) profile in the aqueous humor of both eyes and found significant increase of IL-6, a major proinflammatory cytokine, companied by slight to moderate increases of IL-4, IL-10, TNF-α and IL-17A, which indicated severe intraocular “inflammatory storm” in this IgG4-ROD case.

During treatment, ocular inflammation was effectively suppressed, and the levels of these cytokines were gradually restored. During the dynamic accumulation of the subconjunctival fluid after treatment with UBM, IL-6 showed a simultaneous gradual increase, indicating fluctuation of the intraocular inflammatory state. With resolution of the subconjunctival fluid and disappearance of the ciliary mass, the IL-6 level was readjusted to nearly normal according to the aqueous humor cytokine analysis. Our observations suggested that cytokine level detection is another effective tool for estimating intraocular inflammatory activity, monitoring disease progression, and evaluating therapeutic effect in IgG4-ROD.

A high rate of systemic IgG4-RD involvement exists in patients with IgG4-ROD, particularly in bilateral cases. In a retrospective observational case series study, 14% of patients with unilateral IgG4-ROD and 79% of patients with bilateral IgG4-ROD had extra-ophthalmic involvement [18]. The most common sites of extraocular involvement in IgG4-ROD are the salivary glands and lymph nodes [19, 20]. Given the high rate of extra-ophthalmic involvement, systemic examination and imaging (head, neck, chest, abdomen, and pelvis) of new cases of IgG4-ROD are recommended to identify other sites of involvement.

Systemic prednisone is recommended as first-line therapy because a high rate of response to prednisone is a common feature of IgG4-ROD. Immunosuppressive therapy may be required in steroid-sparing or steroid-resistant cases [1]. Ophthalmic diseases also respond well to glucocorticoids in many patients. However, relapses are frequent after tapering or discontinuing therapy and can progress to a chronic state [10]. Additional immunotherapy is necessary in some cases, including the use of cyclophosphamide, rituximab (the most specific biological treatment with high rates of remission), mycophenolate, methotrexate, cyclosporine, or azathioprine [1, 10]. A possible relationship between IgG4-ROD and lymphoma has been reported. Thus, prolonged surveillance of IgG4-ROD patients is advisable [21, 22].

IgG4-ROD can be challenging to diagnose as a new and rare entity, especially in cases of atypical features, such as ocular mass, conjunctival infiltration, uveitis, or scleritis. It should be suspected in any chronic inflammatory ophthalmological manifestation after excluding the more frequent alternative diagnoses. Combined with ocular cytokinome profiles, multimodal imaging can help detect lesions, monitor disease progression, and guide therapeutic strategy.

## Data Availability

The original contributions presented in the study are included in the article. Further inquiries can be directed to the corresponding author.

## Abbreviations

IgG4-ROD: IgG4-related ophthalmic disease
IHC: Immunohistochemistry
BCVA: Best corrected visual acuity
LP: Light perception
KP: Keratic precipitates
AC: Anterior chamber
MRI: Magnetic resonance imaging
UBM: Ultrasound biomicroscopy
CBC: Complete blood Count
ERD: Exudative retinal detachment
